# Policy Issues in Elder Abuse

**DOI:** 10.1093/geroni/igaf122.1481

**Published:** 2025-12-31

**Authors:** Bob Blancato

**Affiliations:** Elder Justice Coalition, Washington, District of Columbia, United States

## Abstract

My work in elder abuse and elder justice has been at the national policy level for almost the entire 50-year history of elder abuse research. This includes more than 10 years as a Subcommittee Staff Director on the House Select Committee on Aging, when the first hearings on elder abuse in the early 1980’s were initiated and held. Since 2003, I have been the National Coordinator of the bipartisan Elder Justice Coalition, which led the advocacy efforts on behalf of the landmark Elder Justice Act of 2010. Research in elder abuse and its proper dissemination has and continues to make important contributions to both legislative and administration policy in elder justice. This brief presentation will consider what has been achieved in the policy domain relating to elder abuse, both past and present. It will explore initiatives to date on elder justice and other programs concerned with elder abuse, neglect and exploitation. The aim is to identify the relevant strengths and weaknesses/challenges of approaches and initiatives already taken, both past and present and to offer suggestions for future improvements and potential research to support this important area of public policy.

